# Interrelation between gut microbiota, SCFA, and fatty acid composition in pigs

**DOI:** 10.1128/msystems.01049-23

**Published:** 2023-12-14

**Authors:** Cristina Sebastià, Josep M. Folch, Maria Ballester, Jordi Estellé, Magí Passols, María Muñoz, Juan M. García-Casco, Ana I. Fernández, Anna Castelló, Armand Sánchez, Daniel Crespo-Piazuelo

**Affiliations:** 1 Plant and Animal Genomics, Centre for Research in Agricultural Genomics (CRAG), CSIC-IRTA-UAB-UB Consortium, Bellaterra, Spain; 2 Departament de Ciència Animal i dels Aliments, Facultat de Veterinària, Universitat Autònoma de Barcelona (UAB), Bellaterra, Spain; 3 Departament de Genètica i Millora Animal, Institut de Recerca i Tecnologia Agroalimentàries (IRTA), Caldes de Montbui, Spain; 4 Université Paris-Saclay, INRAE, AgroParisTech, GABI, Jouy-en-Josas, France; 5 Departamento de Mejora Genética Animal, INIA-CSIC, Madrid, Spain; 6 Centro I+D en Cerdo Ibérico INIA-Zafra, Zafra, Spain; The University of Maine, Orono, Maine, USA

**Keywords:** gut microbiome, pig, lipid metabolism, prevotella, SCFA, functional prediction

## Abstract

The gut microbiota is a key player in the host metabolism. Some bacteria are able to ferment non-digestible compounds and produce short-chain fatty acids that the host can later transform and accumulate in tissue. In this study, we aimed to better understand the relationships between the microorganisms and the short-chain fatty acid composition of the rectal content, including the possible linkage with the fatty acid composition in backfat and muscle of the pig. We studied a Duroc × Iberian crossbred population, and we found significant correlations between different bacterial and archaeal genera and the fatty acid profile. The abundance of n-butyric acid in the rectal content was positively associated with *Prevotella* spp. and negatively associated with *Akkermansia* spp., while conversely, the abundance of acetic acid was negatively and positively associated with the levels of *Prevotella* spp. and *Akkermansia* spp., respectively. The most abundant genus, *Rikenellaceae* RC9 gut group, had a positive correlation with palmitic acid in muscle and negative correlations with stearic acid in backfat and oleic acid in muscle. These results suggest the possible role of *Prevotella* spp. and *Akkermansia* spp. as biomarkers for acetic and n-butyric acids, and the relationship of *Rikenellaceae* RC9 gut group with the lipid metabolism, building up the potential, although indirect, role of the microbiota in the modification of the backfat and muscle fatty acid composition of the host.

**IMPORTANCE:**

The vital role of the gut microbiota on its host metabolism makes it essential to know how its modulation is mirrored on the fatty acid composition of the host. Our findings suggest *Prevotella* spp. and *Akkermansia* spp. as potential biomarkers for the levels of beneficial short-chain fatty acids and the possible influence of *Rikenellaceae* RC9 gut group in the backfat and muscle fatty acid composition of the pig.

## INTRODUCTION

Pig is one of the most important livestock species, with pork being the second most produced meat worldwide after chicken (1). In Spain, the Iberian pig is an autochthonous breed, usually raised in “dehesas” (holm oaks) and fed with acorns as part of its diet (2). The Iberian pig has lower growth efficiency and higher fat deposition than conventional breeds, but it excels in meat quality due to its different fatty acid (FA) profile. Iberian pork has higher percentage of oleic acid and saturated fatty acid (SFA), and less abundance of polyunsaturated fatty acids (PUFAs) (3, 4), which makes this meat ideal for cured products and appeals to the consumer due to its organoleptic properties. In production farms, Iberian pigs are crossbred with Duroc pigs, producing more efficient animals without overly impairing the production of ham and other cured products (2, 5).

Aside from its interest in meat production, the pig, as a monogastric omnivorous animal, can be used as model species for different human syndromes and conditions, including gut microbiota-related diseases and dietary modulation of the gut population (6, 7). Thus, gut microbiota composition influences host metabolism and its general welfare (8) and is deeply modulated by the diet (9, 10), albeit being also affected by environmental factors and by the host genetics (11–13). During the past decades, distinction and classification of gut microbial communities have been performed through 16S rRNA gene sequencing among other methods (14, 15). In addition, the metabolites produced by the gut microbiota have also been studied. Gut microbiota is able to ferment non-digestible compounds such as dietary fibers and produce, among other metabolites, short-chain fatty acids (SCFAs) (16). In the gut, SCFAs are an energy source available for the host (17) and can be incorporated in different metabolic pathways (18, 19). SCFAs that cross the intestinal barrier and are incorporated into the host metabolism can influence the FA profile across tissues, as they act as a substrate for FA biosynthesis and are also involved in the regulation of the lipid metabolism (20). Hence, identifying the main microbial organisms involved in SCFA production is key to imagine strategies capable of influencing the FA composition of the host through any mechanism able to modify the gut microbiota, such as the diet. Therefore, this study aimed to assess the relationship between gut microbiota, SCFA abundance, and FA profile in backfat and muscle of the pig, which, to our knowledge, has not yet been explored.

## MATERIALS AND METHODS

### Animal material

The animal data for this study consisted of 288 healthy pigs from an F1 Duroc × Iberian crossbred population. All the animals were housed in the same farm under a controlled environment in intensive conditions and were fed *ad libitum* with a standard commercial diet based on barley and wheat. Pigs were slaughtered in a commercial abattoir with an average weight of 138.8 kg (SD = 11.46 kg) in four different days. Samples of backfat, longissimus dorsi muscle, and rectal content were collected, snap-frozen in liquid nitrogen, and preserved at −80°C until further use.

### Microbial DNA extraction and sequencing

Microbial DNA was extracted from 0.2 g of rectal content using the PowerFecal kit (MoBio Laboratories, Carlsbad, CA, USA) and following the manufacturer’s guidelines. DNA purity and concentration were assessed with an ND-1000 spectrophotometer (NanoDrop Technologies, Wilmington, DE, USA).

The V3-V4 region of the 16S rRNA gene was amplified following the recommendations on the *16S Metagenomic Sequencing Library Preparation* guide (Illumina, San Diego, CA, USA). The complete description of the procedure can be accessed at Crespo-Piazuelo et al. (12). In summary, all the amplicon pooled libraries from the 288 animals were sequenced in three runs by a MiSeq System (Illumina) using the MiSeq Reagent Kit v3 (600-cycle format, 2 × 300 bp paired-end reads) in the Sequencing Service of the Fundació per al Foment de la Investigació Sanitària i Biomèdica de la Comunitat Valenciana. A total of 17.991 Gb was obtained with an average of 104,115 reads per sample. An outlier was discarded because it had 1,758,983 reads while the rest of the samples were in a 34,186 to 218,360 range.

### Taxonomy classification

The remaining 287 samples were analyzed with QIIME2 v.2021.11.0 (21). Sequences with a Phred score lower than 33 were filtered out and processed in QIIcentME2 with the DADA2 algorithm, denoising and trimming 280 nucleotides in the forward read sequences and 230 in the reverse read sequences. Two of the samples were discarded because they did not match the quality criteria. The three technical replicates were merged per individual using the sum overlap method. For the 285 remaining samples, phylogeny was calculated and they were taxonomically classified at 99% of similarity using the SILVA database release 138 (22) specifying the V3-V4 region forward and reverse primers. After the QIIME2 pipeline, 46,284 amplicon sequence variants (ASVs) were obtained and filtered with the following procedure to avoid artifacts and singletons. Using R v4.1.3 software (23), we kept only those ASVs with a greater abundance than 0.005% of the total number of counts:


$$\frac{sample\;ASVs\times 100}{\sum ASVs}>0.005$$

Hence, 2,671 ASVs were obtained and grouped in 119 genera, 67 families, and 17 phyla. For diversity analyses, filtered samples were rarefied with phyloseq v.1.38.0 (24) to an equal number of ASVs per individual (*n* = 8,864), which was the minimum number of counts. Then, α- and β-diversities were calculated with phyloseq and vegan v.2.5–7 R packages (25). Thus, α-diversity was estimated by the Shannon index, while β-diversities were determined by dissimilarities of Bray-Curtis (26).

### Fatty acid composition

Fatty acid composition in backfat and longissimus dorsi muscle was measured by gas chromatography. For backfat, the analysis was performed at the Asociación Interprofesional del Cerdo Ibérico at Zafra (Spain) and following the official method explained in the Boletín Oficial del Estado (BOE) (27). Two PerkinElmer chromatographs equipped with a flame ionization detector (FID) were used, with autosamplers and a fused silica capillary column (20-m × 0.32-mm internal diameter and 0.25-µm film thickness). Intramuscular fatty acid composition was performed at Servicio de Técnicas Aplicadas a la Biociencia from Extremadura University, where lipids were extracted from muscle according to Bligh and Dyer (28) and analyzed by gas chromatography using a Bruker Scion 456 GC equipped with an FID and a DB-225ms (Agilent Technologies, Santa Clara, CA, USA) capillary column (30-m × 0.25-mm internal diameter and 0.25-µm film thickness). Results were expressed as the percentage of total fatty acids identified. Out of the total 285 samples, 14 samples were discarded because there was not enough material for measuring FA composition in one of the two tissues. Thus, downstream analyses were carried out with 271 samples. Information on the 16 measured FAs and the 3 calculated indices is shown in Table 1.

**TABLE 1:** Mean relative abundances and SDs^*a*^ of the analyzed fatty acids from backfat and longissimus dorsi muscle

Group	Trait	Backfat		Longissimus dorsi	
		Mean	SD	Mean	SD
SFA^*b*^	C14:0 (myristic acid)	1.57	0.140	1.52	0.147
	C15:0 (pentadecanoic acid)	0.03	0.006	0.05	0.034
	C16:0 (palmitic acid)	24.94	0.888	27.67	1.986
	C17:0 (margaric acid)	0.20	0.037	0.14	0.102
	C18:0 (stearic acid)	10.71	0.834	12.46	1.276
	C20:0 (arachidic acid)	0.20	0.023	0.18	0.108
	Total SFA	37.67	1.419	42.02	3.067
MUFA^*c*^	C16:1 (palmitoleic acid)	2.85	0.482	4.62	0.628
	C17:1 (heptadecenoic acid)	0.25	0.038	0.18	0.088
	C18:1 (oleic acid)	49.11	1.419	47.75	3.276
	C20:1 (gondoic acid)	1.28	0.158	0.67	0.231
	Total MUFA	53.49	1.266	53.23	3.196
PUFA^*d*^	C18:2 (linoleic acid)	7.64	0.691	3.81	0.758
	C18:3 (α-linolenic acid)	0.43	0.043	0.14	0.037
	C20:2 (eicosadienoic acid)	0.46	0.047	0.10	0.097
	C20:3(n-3) (eicosatrienoic acid)	0.06	0.018	0.07	0.054
	C20:3(n-6) (dihomo-γ-linolenic acid)	0.11	0.041	0.08	0.116
	C20:4 (arachidonic acid)	0.14	0.025	0.55	0.291
	Total PUFA	8.84	0.751	4.75	1.045

^*a*^ SD, standard deviation.

^*b*^ SFA, saturated fatty acid.

^*c*^ MUFA, monounsaturated fatty acid.

^*d*^ PUFA, polyunsaturated fatty acid.

### Short-chain fatty acid composition in the rectal content

The abundance of SCFAs in the rectal content was assessed through the analytical method SM 5560-D, using a Varian CP-3800 gas chromatograph with an FID detector and a TRB-FFAP (free fatty acid phase) chromatographic column (15 m × 0.53 mm × 0.5 mm). The FFAP stationary phase was a nitroterephthalic-acid-modified polyethylene glycol (PEG) column. SCFA composition of the 271 individuals is available in Table 2. In addition, principal component analysis of the SCFA composition was performed with the prcomp function in R.

**TABLE 2:** Mean relative abundances and SDs^*a*^ of the analyzed short-chain fatty acids from rectal content

SCFA	Mean	SD
Acetic acid	59.23	2.991
Propionic acid	19.88	1.723
n-Butyric acid	10.13	2.025
iso-Butyric acid	3.11	0.531
n-Valeric acid	2.84	0.347
iso-Valeric acid	4.76	0.682

^*a*^ SD, standard deviation.

### Correlation and association analyses between microbiota, SCFA, and FA compositions

Pearson correlations were calculated within and between the traits from the three different data sets, i.e., microbiota using bacterial genera abundances, SCFA, and FA compositions, using the corr.test function from psych v.2.1.9 R package (29). Thereafter, Benjamini and Hochberg method (30) was applied for multiple testing correction. The significance threshold was established at p.adj <0.05. Correlation results were plotted as heatmaps with the Pheatmap R package v.1.0.12 (31).

In addition, the relationship between the taxonomic data, FA, and SCFA compositions in the 271 samples was studied with the mixOmics v.6.18.1 R package (32, 33). The two data sets used as input were (i) the 38 FA phenotypes (16 backfat FAs, 16 muscle FAs, and 6 SCFAs) pre-corrected by sex (two categories, male and female) and batch (four categories, based on the slaughter day), and (ii) the filtered taxonomic data at genus level, which was normalized using the centered log-ratio transformation option from mixOmics. Then, data were analyzed by two different methods in mixOmics: the regularized canonical correlation analysis (rCCA), using the shrinkage method (λ_1_ = 0.28, λ_2_ = 0.15), and the partial least squares (PLS) method.

Furthermore, the adonis2 function from the vegan R package was used to implement a permutational multivariate analysis of variance (PERMANOVA) analysis for each phenotype, related with the ASV relative abundance table. A non-metric multidimensional scaling (NMDS) using Bray-Curtis dissimilarities was carried out to plot the results, together with an ordination diagram, using vegan’s package different functions.

### Functional prediction analysis

PICRUSt2 (34) plug-in for QIIME2 v.2021.11 was used to carry out the functional prediction analysis using the “full-pipeline” option and the default parameters to obtain the results. The Kyoto Encyclopedia of Genes and Genomes (KEGG) orthology abundances were then processed with R; we calculated the Pearson correlations with Benjamini and Hochberg multiple testing correction, and we performed an association with the FA composition and with the microbial genera through the rCCA method from mixOmics.

## RESULTS

### Taxonomic classification and diversity analysis of gut microbiota

We found 2,671 ASVs in the rectal content of 285 pigs, which were classified in 119 genera, 67 families, and 17 phyla. At phylum level, 2 out of the 17 phyla belonged to the archaea domain, Euryarchaeota and Thermoplasmatota. There was only one genus within each of these two archaeal phyla, *Methanobrevibacter* and an uncultured Methanomethylophilaceae genus, respectively.

**Fig 1:**
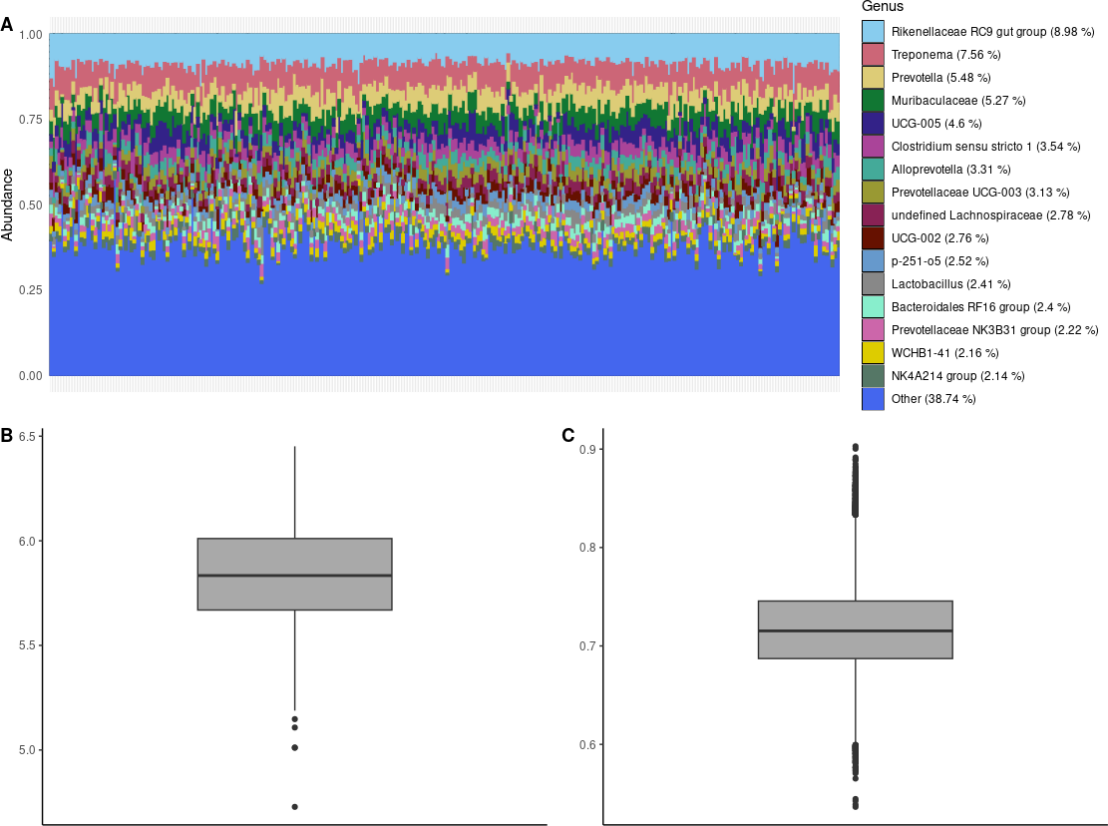
Population diversity in the 285 analyzed samples from rectal content. (A) Stacked barplot for the relative abundance of genera. Genera with a total relative abundance lower than 2% are grouped as “other.” (B) Shannon’s α-diversity in the 285 samples. (C) Bray-Curtis dissimilarities between samples.

The two most abundant phyla were Bacteroidota (44.08%) and Firmicutes (38.17%) (Fig. 1A). Other remarkable phyla in terms of abundance were Spirochaetota (8.18%) and Verrucomicrobiota (3.51%). At genus level, *Rikenellaceae* RC9 gut group (8.98%) was the most abundant, closely followed by *Treponema* (7.56%), *Prevotella* (5.48%), and an unspecified *Muribaculaceae* genus (5.27%). The total counts and the relative abundances at phylum and genus levels are available in Table S1.

To evaluate the richness and evenness of the gut microbiota, α-diversity was calculated using the Shannon index. The average value of the α-diversity was 5.82, ranging from 4.73 to 6.45 (Fig. 1B). The differences between samples were computed with the Bray-Curtis dissimilarities. On average, the β-diversity value was 0.72, ranging from 0.54 to 0.9 (Fig. 1C). After the rarefaction, the diversity values barely changed; the mean α-diversity was 5.8 (4.72–6.39) and the mean β-diversity was 0.73 (0.54–0.91).

### Phenotypic analysis

**Fig 2:**
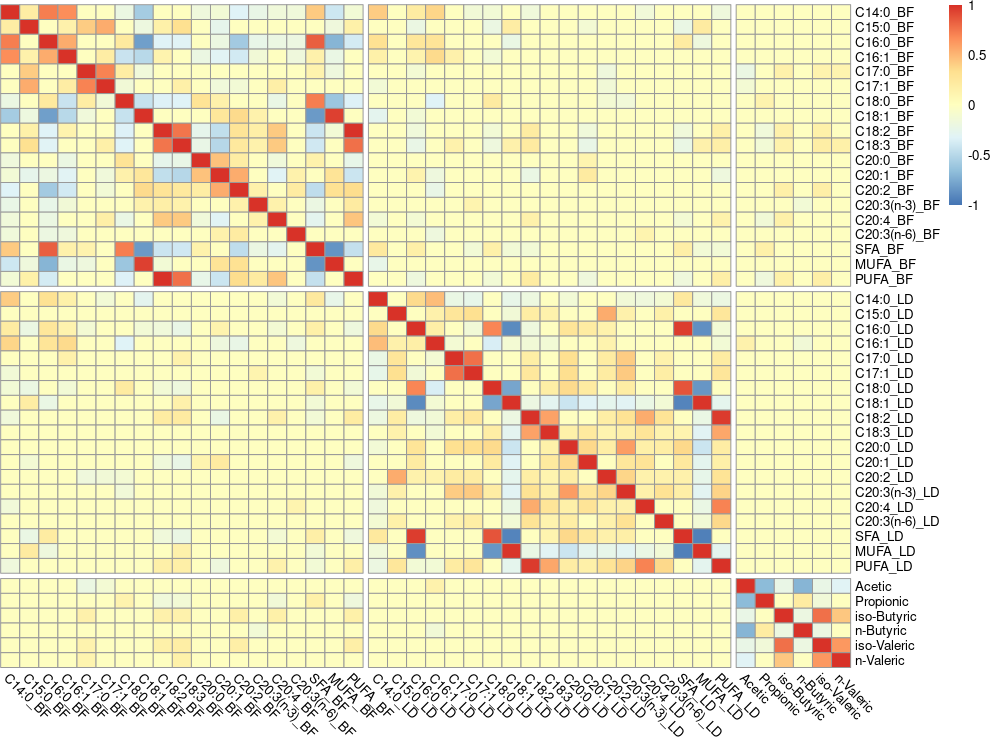
Heatmap of Pearson correlations between fatty acids in backfat, muscle, and rectal content. “BF” extension means that the FA belongs to backfat and “LD” to longissimus dorsi. Acetic, propionic, iso- and n-butyric, and iso- and n-valeric acids come from rectal content. Significant correlations were considered with a *P* value of <0.05. Non-significant correlations were set as 0.

Pearson correlations between tissular FA and rectal SCFA compositions were analyzed in 271 samples. The results of such correlations can be found in Table S2 and are represented as a heatmap in Fig. 2. In general, the abundance of SFA was strongly correlated with the abundance of monounsaturated fatty acid (MUFA) in both backfat and muscle (ρ = −0.85 and ρ = −0.94, respectively), while MUFA and PUFA had only a slight negative correlation in muscle (ρ = −0.27). In backfat, PUFA had a strong negative correlation (ρ = −0.45) with SFA. However, the unsaturated FAs (C16:1, C17:1, C20:1, C20:2, C20:3(n-3), C20:3(n-6), and C20:4) had a positive correlation with their respective SFA (C16:0, C17:0, and C20:0). The only exceptions were the stearic acid (C18:0) and its unsaturated forms (C18:1, C18:2, and C18:3), which showed negative correlations.

Acetic acid was found negatively correlated with all the other SCFAs, especially with propionic and n-butyric acids (ρ = −0.67 and ρ = −0.70, respectively), while propionic and n-butyric acids had a slight positive correlation between them (ρ = 0.20).

### Phenotype and taxonomy association

**Fig 3:**
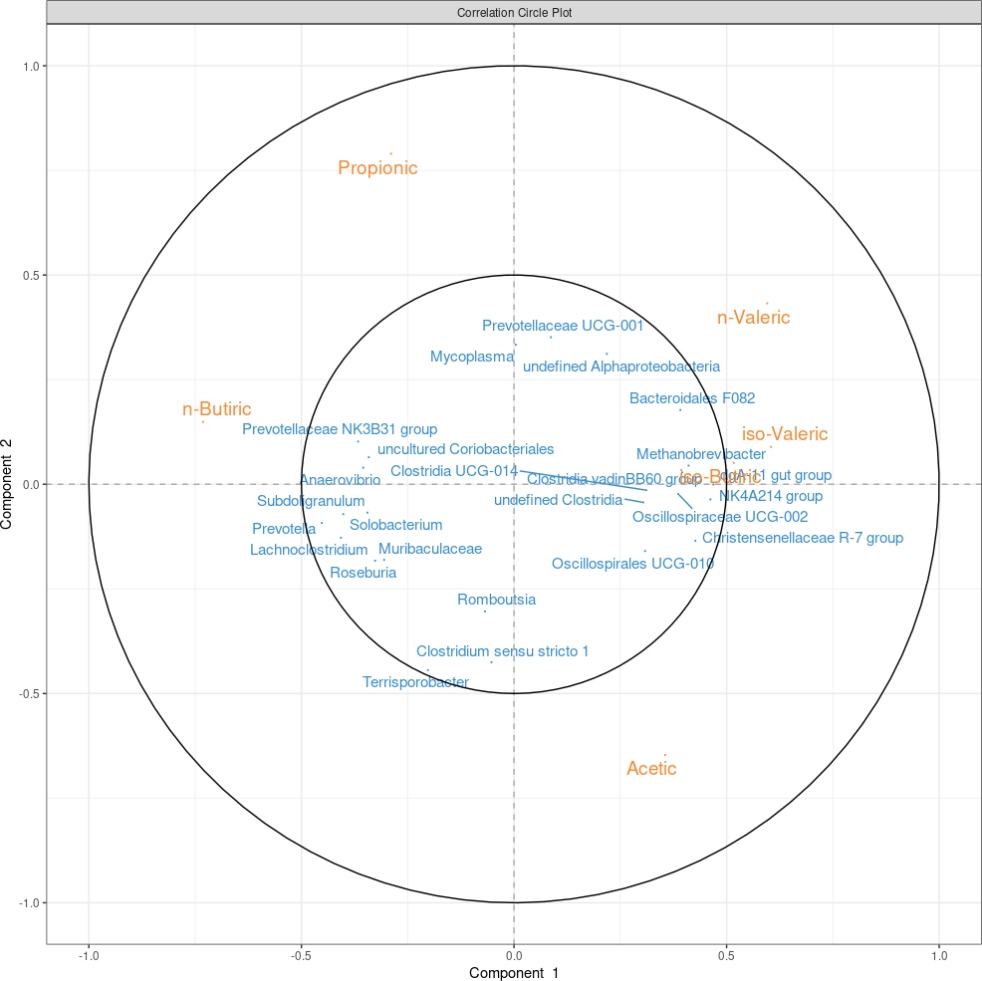
Correlation circular plot between the relative abundance of short-chain fatty acids and bacterial and archaeal genera. For clarity purposes, only variables with a component value greater than |0.3| are represented.

The rCCA method from mixOmics was used to assess the relationship between the rectal microbiota at genus level from the 271 samples with complete data and their rectal SCFA and FA compositions in backfat and muscle. Figure 3 shows a circular plot of the microbial genera and the FA with the strongest correlations (regarding the components), showing only the variables with a component correlation >|0.3|. Globally, the tissular FAs had weaker correlations with the microbiota than the SCFAs, which showed stronger correlations. The n-butyric acid had a clear positive correlation with the bacterial genera *Prevotellaceae* NK3B31 group, *Anaerovibrio*, *Oscillospiraceae* UCG-005, an uncultured *Coriobacteriales*, *Subdoligranulum*, *Prevotella*, *Lactobacillus*, and *Solobacterium*, while the acetic acid was negatively correlated with the same genera. The two most abundant SCFAs, acetic and propionic, had lower correlations with the microbiota, and the acetic acid was placed on the opposing side to propionic and n-butyric acids, showing a negative correlation that can be also appreciated in Fig. 2. After Pearson correlation tests and Benjamini and Hochberg correction, the strongest positive correlations were found between the levels of n-butyric acid and *Prevotella* (ρ = 0.47), the iso-valeric acid and *Oscillospiraceae* UCG-002 (ρ = 0.38), and the n-valeric acid and archaea of the *Methanobrevibacter* (ρ = 0.35) genus. On the other hand, the n-butyric acid also had the strongest negative correlations with *Clostridia vadin* BB60 group (ρ = −0.36), *Akkermansia* (ρ = −0.36), and *Oscillospiraceae* NK4A214 group (ρ = −0.35). Albeit weaker than those reported for the previous SCFAs, the strongest positive correlations with the acetic acid were with *Akkermansia (ρ* = 0.24), *Christensenellaceae* R-7 group (ρ = 0.23), and *Escherichia-Shigella* (ρ = 0.23), while the strongest negative correlations were found with *Prevotella* (ρ = −0.24) and *Prevotellaceae* NK3B31 group (ρ = −0.24). It is also worth noting that the strongest positive correlations with propionic acid were with *Prevotellaceae* UCG-001 (ρ = 0.26) and *Alloprevotella* (ρ = 0.22), and the strongest negative correlations were with *Christensenellaceae* R-7 group (ρ = −0.28), *Oscillospirales* UCG-010 (ρ = −0.24), and *Clostridium sensu stricto* 1 (ρ = −0.23). All the correlations between the microbial genera and the FA composition can be found in Table S3.

There were genera correlated with the six SCFAs: *Prevotellaceae* NK3B31 group and uncultured *Coriobacteriales* were positively correlated with propionic and n-butyric acid and negatively correlated with acetic, iso-butyric, iso-valeric, and n-valeric acids. On the other hand, *Christensenellaceae* R-7 group, *Oscillospiraceae* NK4A214 group, and *Oscillospirales* UCG-010 were positively correlated with acetic, iso-butyric, iso-valeric, and n-valeric acids and negatively correlated with propionic and n-butyric acids.

Regarding the FA composition in tissue, *Rikenellaceae* RC9 gut group had the strongest correlations with some of the most abundant FAs. In backfat, *Rikenellaceae* RC9 gut group had a negative correlation with stearic acid (ρ = −0.22) but showed a positive correlation (ρ = 0.17) with palmitic acid and a negative correlation with oleic acid (ρ = −0.18) in muscle.

**Fig 4:**
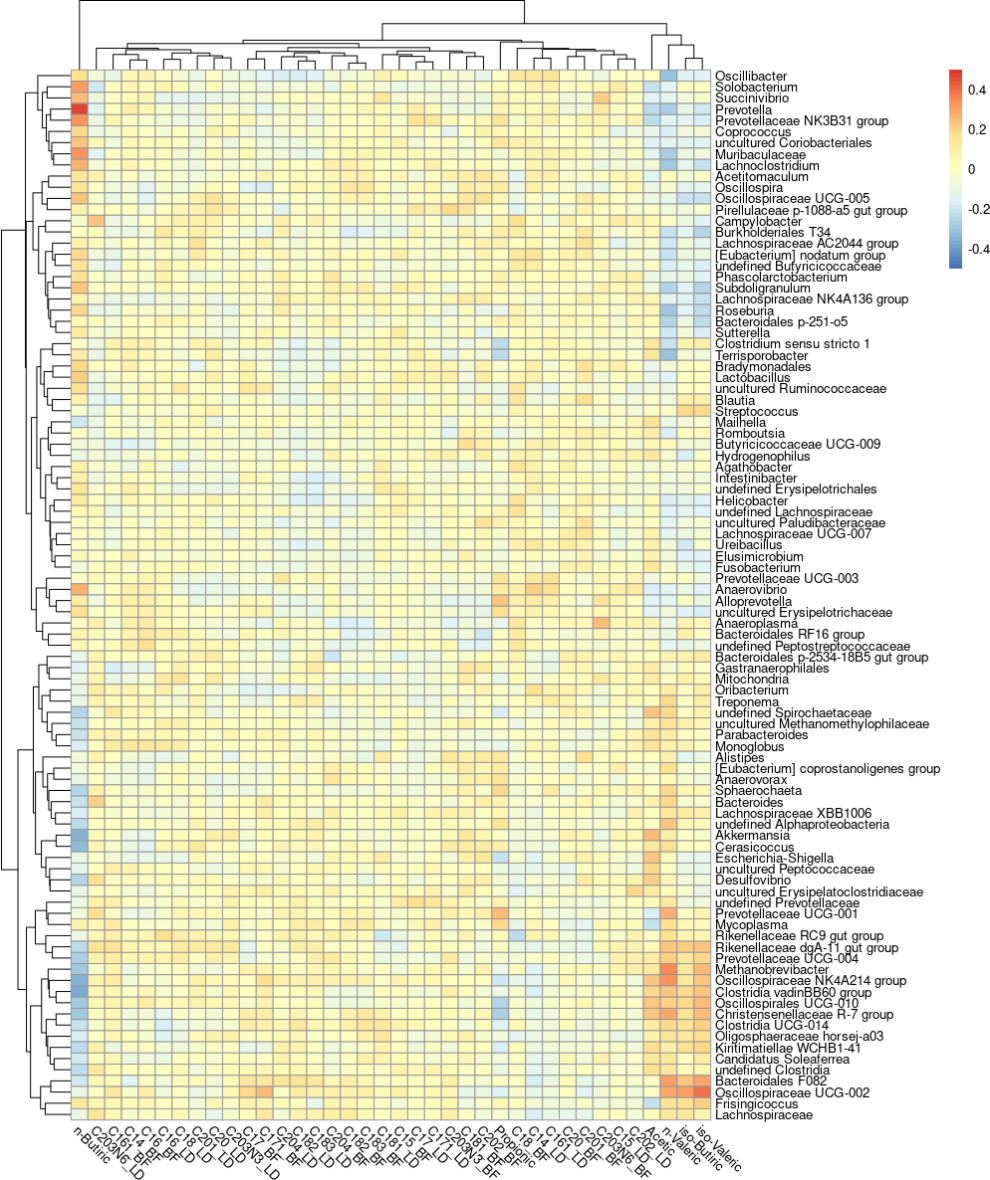
Heatmap for the significant correlations between genera and fatty acid composition in backfat (BF) and muscle (LD) and short-chain fatty acid composition in the rectum. Significant correlations were considered at ρ > |0.15| and *P* value of <0.05. Non-significant correlations were set as 0.

Figure 4 shows the significant correlations (ρ >|0.15| and *P* value < 0.05) between the FA phenotypes and the microbial genera. These microbial genera were also correlated with each other, finding the strongest negative correlation between *Prevotella* and *Oscillospiraceae* NK4A214 group (ρ = −0.54) and the most positive one between *Ureibacillus* and *Hydrogenophilus* (ρ = 0.92). A heatmap with the correlations between all the genera can be found in Supplemental Material S4.

PLS was conducted to find putative predictors for each FA abundance; its results indicate the relationship between genera and each specific FA by assigning a positive or negative weight to each genus to represent their contribution to each phenotype. The results of the PLS analysis carried out independently for each FA phenotype were in agreement with Pearson’s correlations and those reported by the rCCA method. Table S5 shows the top 10 most contributing microbial genera for each FA. *Prevotella* was the second main negative contributor to acetic acid and the main positive contributor to n-butyric acid, while *Akkermansia* was the main positive contributor to acetic acid and the second main negative contributor to n-butyric acid.

**Fig 5:**
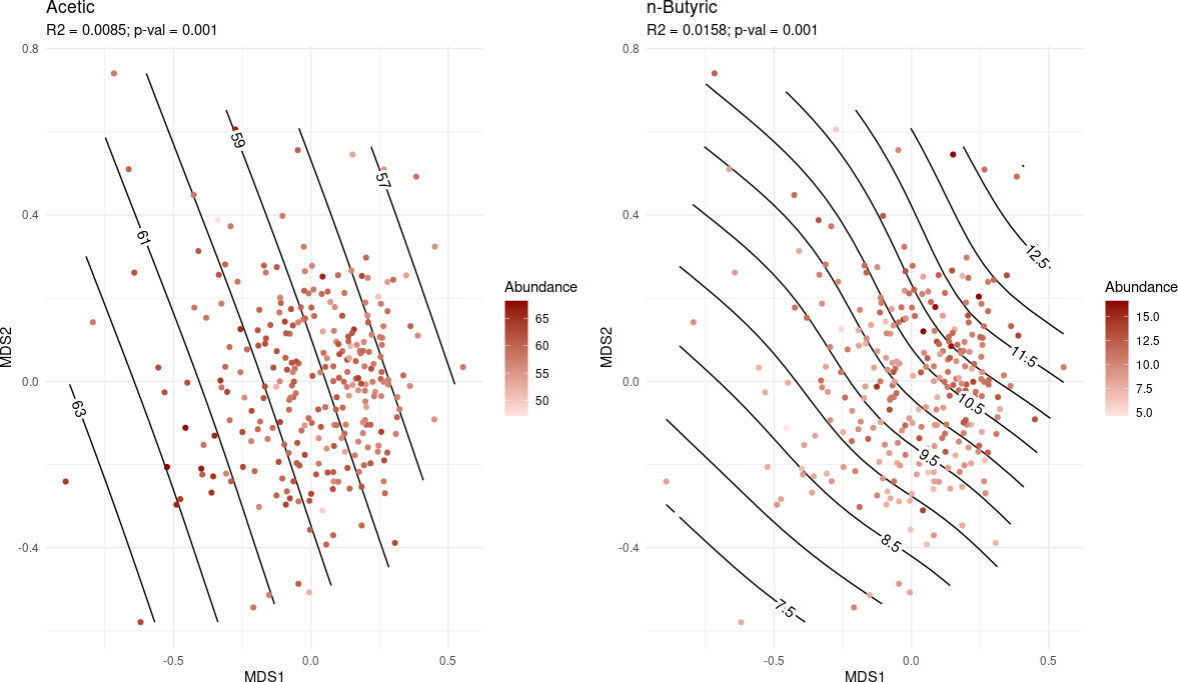
NMDS plots for acetic acid (left) and n-butyric acid (right) with the fatty acid abundances and *R*^2^ from PERMANOVA results. Color saturation represents the SCFA relative abundance on each sample. Black lines show the estimated distribution of the SCFA relative abundances regarding the ordination of the samples based on their β-diversity values (Bray-Curtis dissimilarities). The less curved and the more parallel straight lines are represented, the higher the relationship between the FA relative abundance and the microbiota diversity can be assumed.

The NMDS analysis with the ASV relative abundances reflected a clear relationship between the ordination of the samples based on their β-diversity values and the relative abundances of acetic and n-butyric acids (Fig. 5). The PERMANOVA results found out that a small percentage of the β-diversity was explained by the abundance of these SCFAs, 0.85% for acetic acid and 1.58% for n-butyric acid. Regarding the FA composition in backfat and muscle, the results reported a clear relationship between the arachidonic acid in both tissues and the ordination of the samples. NMDS plots with suggestive PERMANOVA results (*P* value < 0.1) can be accessed in Supplemental Material S6.

### Functional prediction

**Fig 6:**
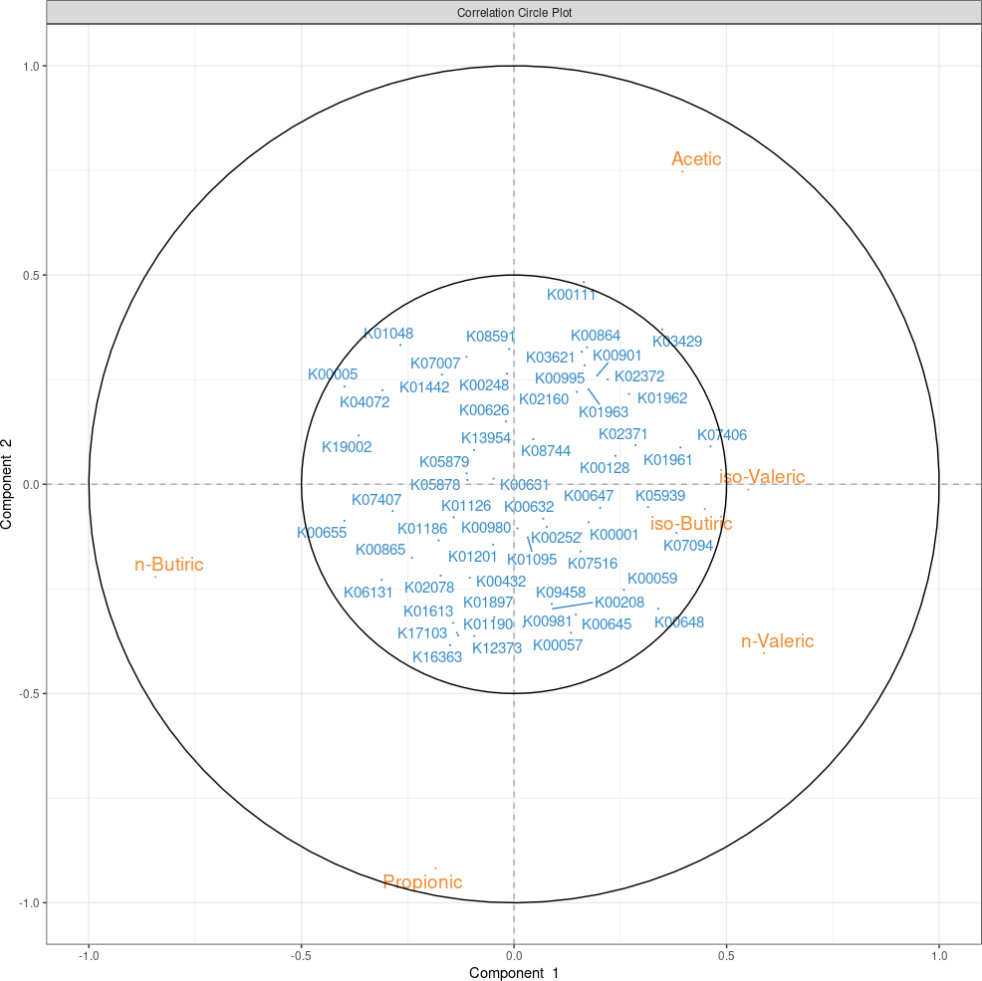
Correlation circular plot between the relative abundance of the short-chain fatty acids and the KEGG orthologs related to lipid metabolism.

From the 2,025 KEGG orthologs (KOs) found with PICRUSt2, 60 were related with lipid metabolism, according to the KEGG Orthology hierarchy. Propionic acid had the highest number of significant correlations with the lipid metabolism KOs; 44 out of the total 60 were significant, while acetic acid and n-butyric acid had 32 and 28 significant correlations, respectively. The strongest positive correlation of acetic acid was with K00111 (ρ = 0.43), while the strongest negative one was with K06131 (ρ = −0.3). For propionic acid, the strongest correlation was with K16363 (ρ = 0.38), while the most negative one was with the K00111 (ρ = −0.42), the same KO as the most positively correlated with acetic acid. In n-butyric acid, the highest positive correlation was with K00655 (ρ = 0.34), while the strongest negative one was with K07406 (ρ = −0.40). In n-valeric acid, the highest correlation was with K00648 (ρ = 0.34), and the most negative one was with K01048 (ρ = −0.37). Iso-butyric and iso-valeric acids had weaker correlations than |0.3|. In general, the negative correlations were stronger than the positive ones. Figure 6 shows the rCCA results between the SCFAs and the KOs related with lipid metabolism. This plot highlights the pattern of the correlations between the KOs and the SCFAs, which follows opposite directions for acetic versus propionic and n-butyric acids, similarly to the taxonomic results. In this plot, there are two KOs that surpass the 0.5 radius threshold, K00111 and K03429, which are two of the KOs with stronger correlations with the three most abundant SCFAs, positive with acetic (ρ = 0.43 and *ρ* = 0.39, respectively), and negative with propionic (ρ = −0.42 and ρ = −0.37, respectively) and n-butyric acids (ρ = −0.25 and ρ = −0.36, respectively).

Similarly to the previous results, tissular FAs had weaker correlations with KOs and less significant results. Only 9 FAs (out of 32), 8 of backfat (pentadecanoic, margaric, heptadecenoic, stearic, eicosadienoic, eicosatrienoic, dihomo-γ-linolenic, and arachidonic acids) and palmitoleic acid from muscle had stronger correlations than |0.2|, most of them unclassified, poorly characterized, or KOs belonging to different protein families. Table S7 shows the significant correlations between the FA composition and the KEGG Orthology results.

KO correlations with microbial genera had higher and more significant values. The highest correlation was found between *Treponema* and K00209 (ρ = 0.71), while the strongest negative correlation was found between the genus *Bacteroidales* F082 and K00655 (ρ = −0.58). Supplemental Material S8 shows a heatmap of all the significant correlations between the KEGG orthologs and the microbial genera.

## DISCUSSION

In this study, we analyzed the correlation between the microbiota and SCFA composition of the rectal content and backfat and muscle FA composition of Duroc × Iberian finishing pigs. Even though this is not a traditional backcross of Landrace, Pietrain., or Large White breeds, it is a hybrid population used for dry-cured products, and the fact that it is an F1 cross minimizes the genetic effect of an F2 three-way cross or backcross. In addition, the samples were taken at the moment of slaughter and immediately frozen, which gives results that resemble more the reality in the pig gut than taking feces samples in the farm.

In accordance with previous studies about the pig gut microbiota in the distal colon (35–38), Bacteroidota and Firmicutes were the two most abundant phyla in our study, representing 82.25% of the total number of counts. Although Firmicutes was less abundant than Bacteroidota in our study, Firmicutes usually is the most prevalent phylum along the swine gastrointestinal tract, while Bacteroidota only has an increased presence in the colon (39–41). In a previous study of the same population conducted by our research group (12), Firmicutes and Bacteroidota also represented 82.83% of the total number of counts, albeit Firmicutes was reported as the most abundant phylum, probably due to the differences between the databases used for the taxonomic classification (GreenGenes v.13.8 and SILVA138) and the different QIIME versions used (QIIME v.1.9.1 and QIIME2 v.2021.11.0). Other studies, conducted with pigs at different age stages and supplemented with probiotics, also found a higher relative abundance of Bacteroidota versus Firmicutes in feces (42, 43). These differences between studies are difficult to address, as the pigs were not raised in the same conditions, and neither the breeds nor the diets were similar. In addition, the composition of the gut microbiota is dynamic and changes through age and sections (39, 41, 44). A possible explanation for the higher presence of Bacteroidota than Firmicutes in our study may be due to the adaptation of the microbiota to the diet, supported by the fact that three out of the four most abundant genera belonged to the Bacteroidota phylum (*Rikenellaceae* RC9 gut group, *Prevotella*, and an unspecified *Muribaculaceae* genus), whereas the remaining one (*Treponema*) belonged to the Spirochaetota phylum. *Prevotella* is usually reported as one of the most abundant genera in the porcine colon, and it is favored in the gut of pigs and human populations with high-fiber diets (45, 46). In the same manner, the levels of Muribaculaceae decrease in high-fat-fed and trans-fatty-acid-fed obese rats (47), and together with *Rikenellaceae* RC9 gut group, pigs supplemented with a fruit and vegetable diet also had an increase of Muribaculaceae (48).

The strongest correlation found between all the FA phenotypes and the microbiota composition was between the relative abundances of *Prevotella* and n-butyric acid. In agreement with the PLS results, *Prevotella* was the highest positive contributor to the levels of n-butyric acid in the rectal content and the second highest negative contributor to acetic acid levels after *Prevotellaceae* NK3B31 group. Remarkably, *Prevotella* was found to be negatively correlated with acetic acid and with butyrate-producing *Clostridia*. Thus, unsurprisingly, some of these butyrate-producing bacteria were positively correlated with the acetic acid and negatively correlated with the n-butyric acid. Gut butyrate can be produced through four pathways, with one of them having the acetate as the main intermediate product, whereas the other ones use succinate, glutarate, or lysine (49). Although our 16S rRNA gene sequencing method was not designed for the taxonomic classification at species level, the results suggest that *Prevotella* genus is involved in the succinate pathway, hinting that the species present in the samples may be *Prevotella copri* or *P. ruminicola* (50, 51). *P. copri* is the most abundant species of the *Prevotella* genus in the gut of adult pigs (52) and produces succinate as a metabolite, which can be transformed to butyrate through its conversion to butyryl coenzyme A (butyryl-CoA) (53).

Spirochaetota was the third most abundant phylum, mainly due to the abundance of *Treponema. Treponema* has been associated with crude fiber digestibility (44) and feed efficiency (54). This genus plays a role in dietary fiber degradation in finishing pigs (44), which provides energy to the host catabolizing indigestible components (55). The negative correlation found between *Treponema* and *Prevotella* has been already reported in pigs (56, 57), possibly due to the fact that both genera may compete for the degradation of the dietary fiber. However, *Prevotella* genus can break down dietary polysaccharides, such as arabinoxylans from wheat and barley, and produce SCFAs (58), while *Treponema* has an essential role in catabolizing dietary non-digestible components, such as cellulose and lignin (44).

The most abundant taxon at the genus level, *Rikenellaceae* RC9 gut group, was negatively correlated with the stearic acid levels in backfat and the oleic acid levels in muscle, while it was also positively correlated with the content of palmitic acid in muscle. Supporting this finding was the strong negative correlation observed between palmitic and oleic in both tissues. As previously mentioned, *Rikenellaceae* RC9 gut group was negatively correlated with *Prevotella*, whereas it was positively correlated with another member of the same family, *Rikenellaceae* dgA-11 gut group*. Rikenellaceae* RC9 gut group may be involved in fatty acid metabolism, as suggested in reference 59. It has been found in higher abundance in high-fat fed mice than in control animals (60, 61), and it is a dominant genus in the ileum of pigs with low food conversion ratio (62). These results may suggest the negative role of *Rikenellaceae* RC9 gut group in meat quality.

Despite *Akkermansia* not being as abundant as other genera, it presented a positive correlation with acetic acid and a negative correlation with n-butyric acid. The positive correlation with acetic acid can be explained because *A. muciniphila*, the main species of this genus, breaks down the mucin of the colon, producing acetic and propionic acids (63). The PLS results showed *Akkermansia* as the first positive contributor to acetic acid and the second negative contributor to n-butyric acid after *Clostridia vadin* BB60 group. These results for *Akkermansia* were in contraposition to the relationship with acetic and n-butyric acid reported for *Prevotella*; thus, as expected, a negative relationship between *Akkermansia* and *Prevotella* was observed in our rCCA and PLS results. This antagonistic relationship has also been found in certain situations in the human gut microbiome (64, 65). Regarding its potential role, *Akkermansia* belongs to the Verrucomicrobiota phylum and has been related with host health, having a reduced presence in mouse models with metabolic disorders (66). Its negative relationship with obesity development in humans and other species has also been reported (67). In summary, the *Akkermansia* genus could be a biomarker of gut health, as reported in humans (68).

*Methanobrevibacter* (phylum Euryarchaeota) and the uncultured Methanomethylophilaceae (phylum Thermoplasmatota) were the only archaeal genera found, but they were not significantly correlated with each other. These two archaea are methanogens, and although methane increases SCFA production (69), their correlation with all the studied SCFAs was not always positive. Both genera had negative correlations with n-butyric acid and positive correlations with n-valeric acid. In addition, *Methanobrevibacter* was positively correlated with acetic, iso-butyric, and iso-valeric acids. Methanogens from genus *Methanobrevibacter*, such as *Methanobrevibacter smithii*, need acetate to grow (70), which means that in an environment rich in acetate, they will be found in higher abundance.

Regarding the functional analysis, the two KOs related with lipid metabolism with the strongest correlations were K00111 and K03429, both with positive correlations with acetic acid and negative correlations with propionic and n-butyric acids. These two KOs play a role in lipid metabolism, K00111 is the glycerol-3-phosphate dehydrogenase (EC:1.1.5.3), which is involved in glycerophospholipid metabolism. This enzyme can be found in the bacterial cytoplasmic membrane and catalyzes the change from glycerol 3-phosphate to glycerone phosphate and vice versa (71, 72). Glycerol is a constitutive molecule of lipids and glycerophospholipids and is key to phospholipid biosynthesis and carbon metabolism, while glycerone phosphate is involved in glycolysis (73). Glycerol and glycerone phosphate can be fermented and transformed in simpler molecules such as acetate, propionate, or butyrate, which will depend on the environmental conditions and bacteria found (74).

K03429 is the processive 1,2-diacylglycerol beta-glucosyltransferase (EC:2.4.1.315) and is involved in different pathways such as glycerolipid metabolism or teichoic acid biosynthesis. This enzyme catalyzes the reversible transformation from diacylglycerol to monoglucosyldiacylglycerol, from this one to diglucosyldiacylglycerol, which can be transformed into lipoteichoic acid, and from diglucosyldiacylglicerol to triglucosyldiacylglycerol (71, 75). Lipoteichoic acid can be found in the cell wall of Gram-positive bacteria that belong to Firmicutes phylum, such as *Clostridia* (76). Our results showed positive correlations of different *Clostridia* (*Clostridia* UCG-014, undefined *Clostridia*, *Clostridia vadin* BB60 group, and *Clostridium sensu stricto*) with acetic acid and negative correlations with propionic and n-butyric acid, most of them significant, which agrees with this finding in the functional prediction.

On the other hand, K00655 and K16363 showed positive correlations with n-butyric and propionic acids (they were the strongest correlation for the respective SCFA) and negative correlations with acetic acid. K00655 is the 1-acyl-sn-glycerol-3-phosphate acyltransferase (EC:2.3.1.51), which is involved in phosphatidic acid production and, therefore, in cell membrane composition. To our knowledge, there is no previous report of a relationship found between K00655 and butyrate, but interestingly, this KO was the one with the strongest negative correlation with a microbial genus, F082, which is a member of the order Bacteroidales. This genus also had a slight negative correlation with n-butyric acid and no significant correlation with propionic acid, although *Bacteroidales* F082 is considered a propionate producer in rumen (77), but its role in intestinal fermentation is not well defined yet.

K16363 encodes for UDP-3-O-[3-hydroxymyristoyl] N-acetylglucosamine deacetylase/3-hydroxyacyl-[acyl-carrier-protein] dehydratase (EC:3.5.1.108 4.2.1.59). The first enzyme is involved in the first step of the lipopolysaccharide production and, therefore, in the cell membrane of Gram-negative bacteria (78), such as *Phascolarctobacterium*, *Sphaerochaeta*, *Anaerovibrio*, *Bacteroidales* RF16 group, or all the members of the Prevotellaceae family with which the correlations were positive. This KO had negative correlations with Gram-positive bacteria such as *Clostridia*.

*Treponema* and K00209 shared the strongest Pearson correlation. K00209 encodes for enoyl-[acyl-carrier protein] reductase/trans-2-enoyl-CoA reductase (NAD+) (EC:1.3.1.9 1.3.1.44), which is involved in fatty acid biosynthesis and elongation. Trans-2-enoyl-CoA reductase (NAD+) was described for the first time in the microalga *Euglena gracilis* (79), but the first enzymatic characterization of a trans-2-enoyl-CoA reductase was done in *Treponema denticola* (80). This family of proteins is involved in fatty acid biosynthesis, but they work with CoA esters instead of acyl carrier protein (ACP) esters (79).

### Conclusions

In summary, the most abundant phyla found in rectal content were Bacteroidota and Firmicutes, while the most abundant genera, *Rikenellaceae* RC9 gut group, *Treponema*, *Prevotella*, and an unspecified *Muribaculaceae* belonged to the Bacteroidota or Spirochaetota phyla. While some bacteria may be involved in the lipid metabolism of the host, as indicated by the correlations observed between their abundances and the FA composition, specifically the SCFA content in the gut, these relationships were weak maybe due to the indirect and long paths between the two phenotypes. Of note, the levels of *Akkermansia* and *Prevotella* were negatively correlated in our study, and their main positive or negative contribution to acetic and n-butyric acids in the rectal content strengthens their role as potential biomarkers for these two SCFAs. The functional prediction analysis showed coherent results regarding the relationships between the KEGG orthologs and the microbial genera or the SCFA. The most abundant genus, *Rikenellaceae* RC9 gut group, had the highest correlations with stearic, palmitic, and oleic acid levels in backfat and muscle, further indicating the potential, although indirect, role of the microbiota in the modification of the FA composition in tissue. Nonetheless, FA composition is a cumulative trait that is developed and changed throughout the life of the animal, same as the modification of the microbiota that growing animals experiment. Thus, further analyses are warranted to measure the microbiota and the FA composition in different time points to better determine the implications of the microbiota over the FA composition in tissue.

## Data Availability

Raw sequencing data can be accessed under the BioProject accession number PRJNA540380 in the National Center for Biotechnology Information (NCBI) Sequence Read Archive (SRA).

## SUPPLEMENTAL MATERIAL

Material S4 msystems.01049-23-s0001.tiff Heatmap with the correlations between the microbial genera.

Material S6 msystems.01049-23-s0002.tiff PERMANOVA results for fatty acids represented in NMDS plots.

Material S8 msystems.01049-23-s0003.tiff Heatmap of the significant correlations between the KEGG orthologs involved in lipid metabolism and the microbial genera.

Legends msystems.01049-23-s0004.docx Legends for the supplemental material.

Table S1 msystems.01049-23-s0005.xlsx Total counts and relative abundance of taxa at phylum and genus level.

Table S2 msystems.01049-23-s0006.xlsx Correlations between all the fatty acids and their *P*-values.

Table S3 msystems.01049-23-s0007.xlsx Correlations between all the fatty acids and the microbial genera.

Table S5 msystems.01049-23-s0008.docx Top 10 significant correlations between each fatty acid and the microbial genera.

Table S7 msystems.01049-23-s0009.xlsx Correlations between all the KEGG orthologs and the fatty acid composition.

ASM does not own the copyrights to Supplemental Material that may be linked to, or accessed through, an article. The authors have granted ASM a non-exclusive, world-wide license to publish the Supplemental Material files. Please contact the corresponding author directly for reuse.
