# Supplementary figures and images for: Interrelation between gut microbiota, SCFA, and fatty acid composition in pigs
Source: mSystems. 2023 Dec 14;9(1):e01049-23. doi: 10.1128/msystems.01049-23 (PMC10804976; doi:10.1128/msystems.01049-23)

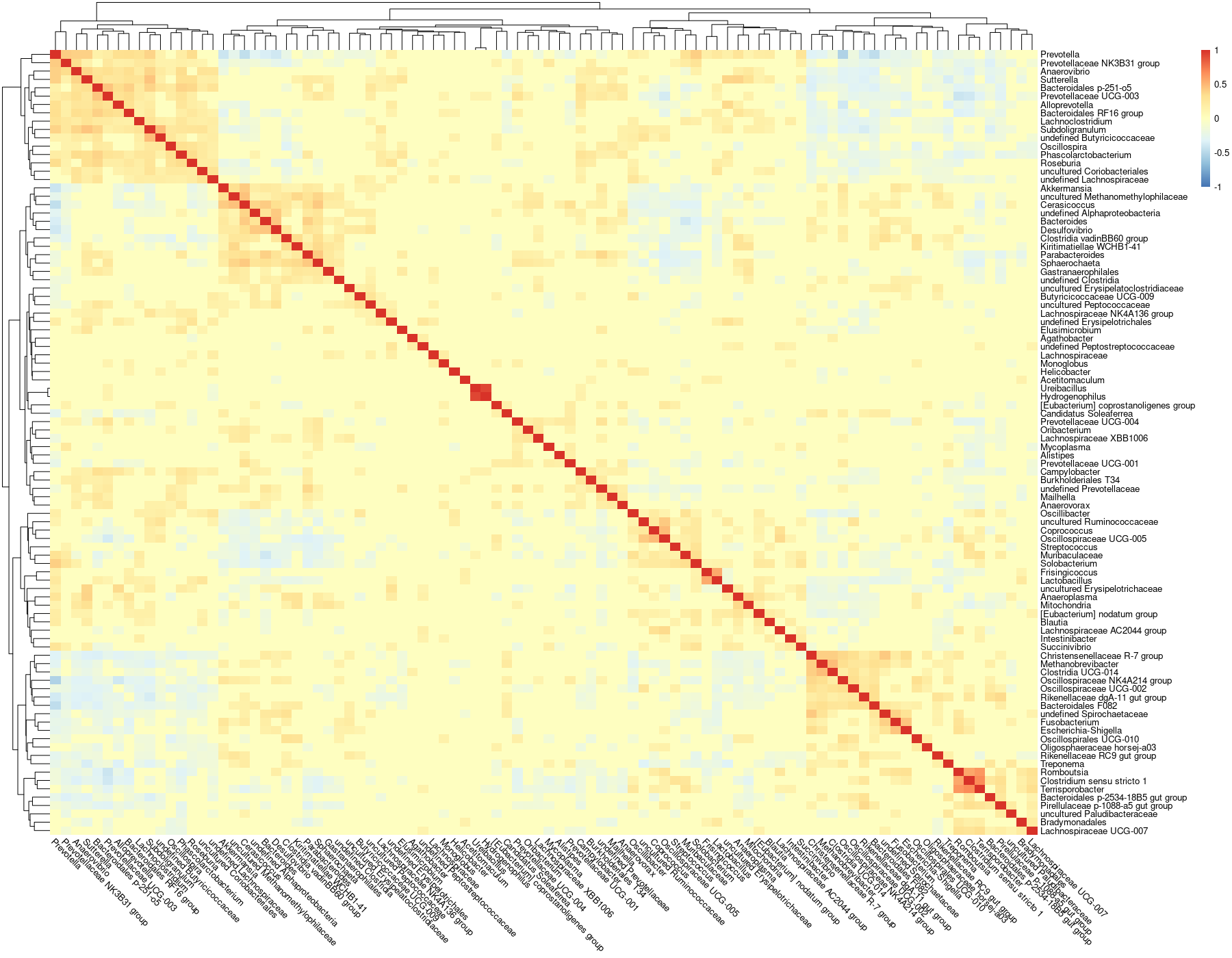

Supplement: Material S4 — Heatmap with the correlations between the microbial genera. [file msystems.01049-23-s0001.tiff]

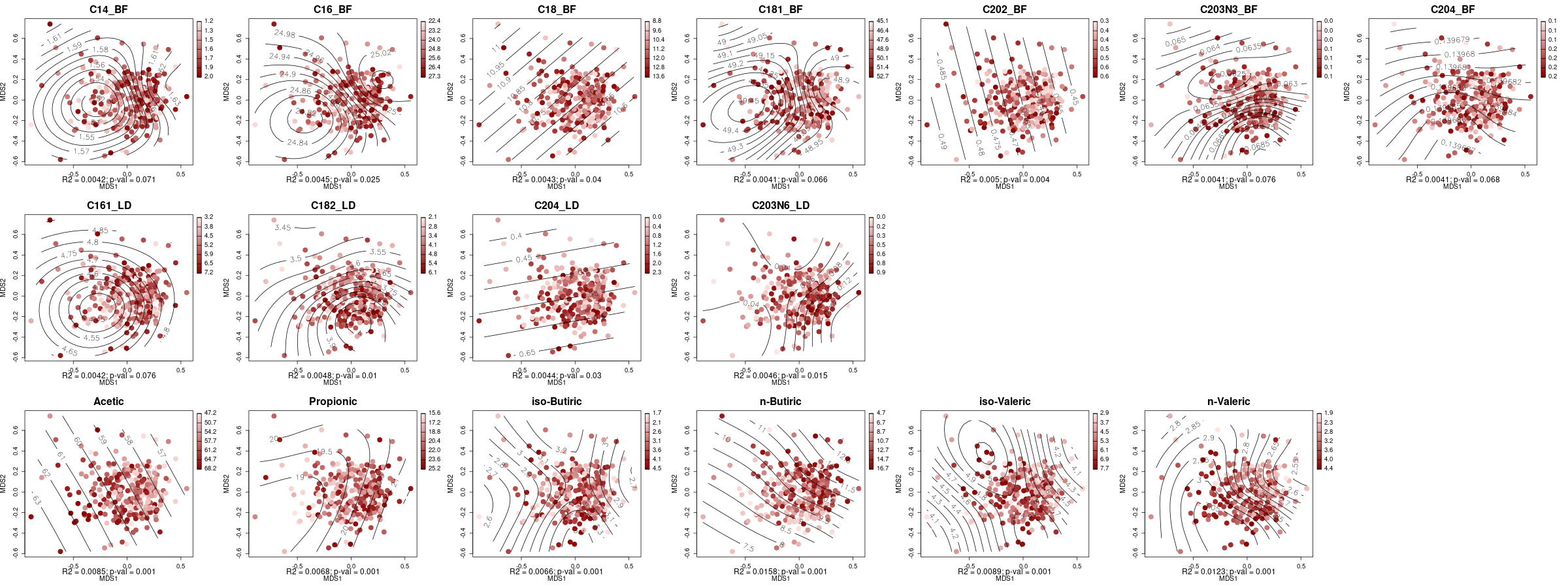

Supplement: Material S6 — PERMANOVA results for fatty acids represented in NMDS plots. [file msystems.01049-23-s0002.tiff]

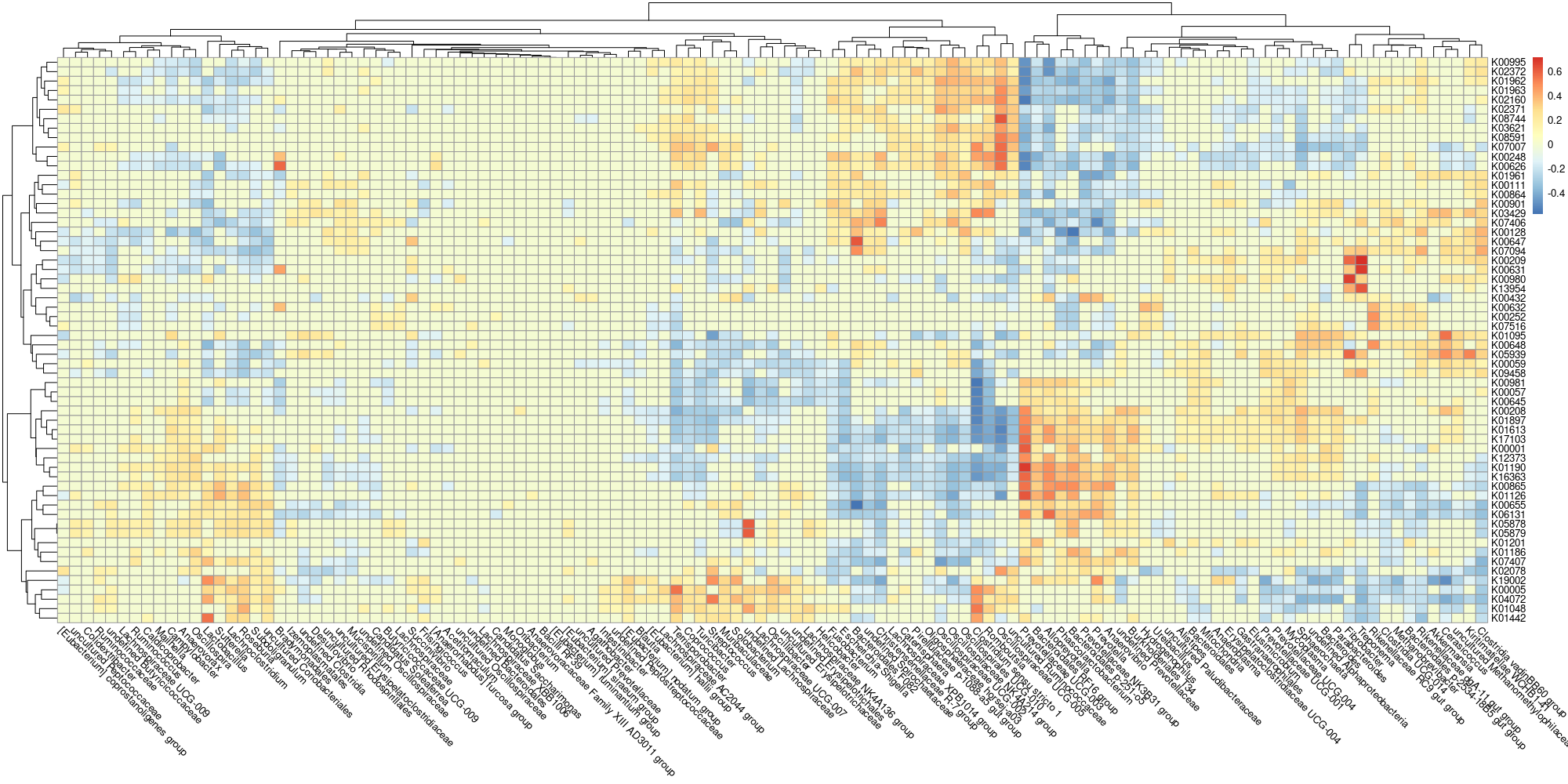

Supplement: Material S8 — Heatmap of the significant correlations between the KEGG orthologs involved in lipid metabolism and the microbial genera. [file msystems.01049-23-s0003.tiff]
