# Supplementary material for: Interrelation between gut microbiota, SCFA, and fatty acid composition in pigs
Source: mSystems. 2023 Dec 14;9(1):e01049-23. doi: 10.1128/msystems.01049-23 (PMC10804976; doi:10.1128/msystems.01049-23)
Supplement: Legends — for the supplemental material. [file msystems.01049-23-s0004.docx]

# **Supplemental Material**

Supplemental Table S1. Total counts and relative abundance of taxa at phylum and genus level. The number of positive samples, mean, standard deviation (SD), minimum and maximum counts are also calculated.

Supplemental Table S2. Correlations between all the fatty acids and their p-values.

Supplemental Table S3. Correlations between all the fatty acids and the microbial genera. P-values are included.

Supplemental Material S4. Heatmap with the correlations between the microbial genera. Non-significant relationships (p-value > 0.05) were set to 0.

Supplemental Table S5. Top 10 significant correlations between each fatty acid and the microbial genera.

Supplemental Material S6. Significant (p-value < 0.05) and suggestive (p-value < 0.1) PERMANOVA results for fatty acids represented in NMDS plots.

Supplemental Table S7. Correlations between all the KEGG orthologs and the fatty acid composition.

Supplemental Material S8. Heatmap of the significant correlations between the KEGG orthologs involved in lipid metabolism and the microbial genera. Non-significant relationships (p-value > 0.05) were set to 0.
