# Supplementary material for: Interrelation between gut microbiota, SCFA, and fatty acid composition in pigs
Source: mSystems. 2023 Dec 14;9(1):e01049-23. doi: 10.1128/msystems.01049-23 (PMC10804976; doi:10.1128/msystems.01049-23)
Supplement: Table S5 — Top 10 significant correlations between each fatty acid and the microbial genera. [file msystems.01049-23-s0008.docx]

Supplemental Table S5. Top 10 significant correlations between each fatty acid and the microbial genera.

|  | Phenotype | Genera |
| --- | --- | --- |
| Backfat | C14:0 | *Butyricicoccaceae UCG-009* (−0.24), *unspecified Ruminococcaceae* (0.21), *Bacteroidales F082* (−0.20), *Monoglobus* (0.20), *Escherichia-Shigella* (−0.17), *Gastranaerophilales* (−0.16), *Prevotellaceae UCG-001* (−0.16), *Bradymonadales* (0.16), *Cerasicoccus* (−0.15), *Anaerovibrio* (0.15) |
|  | C15:0 | *Bacteroidales p-2534-18B5 gut group* (−0.23), *Rikenellaceae dgA-11 gut group* (−0.21), *undefined Prevotellaceae* (0.19), *Anaerorhabdus furcosa group* (−0.19), *Burkholderiales T34* (−0.19), *Sutterella* (0.18), *uncultured Prevotellaceae* (0.18), *Rikenellaceae RC9 gut group* (−0.17), *Subdoligranulum* (0.17), *Fibrobacter* (0.17) |
|  | C16:0 | *Undefined Peptostreptococcaceae* (0.24), *Oscillospiraceae UCG-005* (−0.23), *Oscillospira* (−0.19), *Butyricicoccaceae UCG-009* (−0.19), *Cerasicoccus* (−0.19), *Monoglobus* (0.19), *Candidatus Saccharimonas* (−0.19), *Bacteroidales RF16 group* (0.18), *Anaeroplasma* (0.17), *Lachnospiraceae NK4A136 group* (−0.17) |
|  | C17:0 | *Oscillospiraceae UCG-002* (0.31), *Oribacterium* (−0.24), *Oscillospira* (−0.24), *uncultured Ruminococcaceae* (0.21), *Bacteroidales F082* (0.20), *Rikenellaceae dgA-11 gut group* (−0.19), *Kiritimatiellae WCHB1-41* (−0.19), *Prevotellaceae UCG-004* (−0.19), *Oscillibacter* (−0.17), *uncultured Oscillospiraceae* (0.16) |
|  | C18:0 | *Rikenellaceae RC9 gut group* (−0.27), *Campylobacter* (0.21), *agathobacter* (0.19), *Helicobacter* (0.19), *uncultured Erysipelatoclostridiaceae* (−0.18), *undefined Lachnospiraceae* (0.18), *uncultured Ruminococcaceae* (0.17), *Anaeroplasma* (0.17), *Blautia* (0.17), *Oligosphaeraceae horsej-a03* (−0.17) |
|  | C20:0 | *Uncultured Peptococcaceae* (−0.23), *Catenisphaera* (0.22), *Mycoplasma* (−0.21), *Ureibacillus* (0.20), *unspecified Lachnospiraceae* (−0.19), *Bacteroidales p-2534-18B5 gut group* (0.18), *Lachnospiraceae UCG-007* (0.18), *Desulfovibrio* (−0.17), *Campylobacter* (0.17), *unspecified Ruminococcaceae* (−0.17) |
|  | C16:1 | *Gastranaerophilales* (−0.25), *Rikenellaceae dgA-11 gut group* (0.24), *undefined Butyricicoccaceae* (−0.19), *Blautia* (−0.19), *Butyricicoccaceae UCG-009* (−0.19), *undefined Spirochaetaceae* (0.18), *Oscillospiraceae UCG-002* (0.18), *unspecified Bacteroidales* (0.17), *Monoglobus* (0.17), *Oscillospiraceae NK4A214 group* (0.17) |
|  | C17:1 | *Oscillospiraceae UCG-002* (0.32), *Oscillospiracecae NK4A214 group* (0.23), *Oscillospira* (−0.22), *Oscillibacter* (−0.19), *Prevotella* (−0.18), *Bacteroidales F082* (0.18), *Anaeroplasma* (−0.18), *Lachnospiraceae UCG-009* (−0.17), *Oribacterium* (−0.17), *Burkholderiales T34* (−0.16) |
|  | C18:1 | *Butyricicoccaceae UCG-009* 0.26), *undefined Peptostreptococcaceae* (−0.19), *Oscillospiraceae UCG-005* (0.19), *Candidatus Saccharimonas* (0.19), *Pirellulaceae p-1088-a5 gut group* (0.18), *Akkermansia* (0.18), *unspecified Gastranaerophilales* (0.18), *unspecified Ruminococcaceae* (−0.17), *uncultured Erysipelatoclostridiaceae* (0.17), *Eubacterium coprostalinogenes group* (0.17) |
|  | C18:2 | *Anaeroplasma* (−0.25), *Burkholderiales T34* (−0.25), *Bacteroidales RF16 group* (−0.22), *Oscillospira* (0.20), *Helicobacter* (−0.19), *Anaerorhabdus furcosa group* (−0.18), *Colidextribacter* (−0.18), *unspecified Rikettsiales* (−0.17), *Lachnospiraceae UCG-009* (−0.17), *Elusimicrobium* (−0.16) |
|  | C18:3 | *Burkholderiales T34* (−0.24), *Bacteroidales RF16 group* (−0.24), *Anaeroplasma* (−0.22), *Oscillospira* (0.19), *Clostridia UCG-014* (0.19), *Christensenellaceae R-7 group* (0.19), *Colidextribacter* (−0.18), *Alistipes* (−0.18), *unspecified Rikettsiales* (−0.17), *Helicobacter* (−0.17) |
|  | C20:1 | *Alistipes* (0.24), *Blautia* (0.22), *Lachnospiraceae AC2044 group* (0.21), *Lachnospiraceae UCG-007* (0.21), *uncultured Paludibacteraceae* (0.21), *Oscillospiraceae UCG-002* (−0.20), *Bradymonadales* (0.19), *Mycoplasma* (−0.17), *Burkholderiales T34* (0.16), *Anaerorhabdus furcosa group* (0.16) |
|  | C20:2 | *Bacteroidales RF16 group* (−0.25), *Oscillospiraceae UCG-005* (0.21), undefined Peptostreptococcaceae (−0.21), uncultured Paludibacteraceae (0.20), *Sutterella* (−0.18), *Acetitomaculum* (0.18), *Escherichia-Shigella* (0.17), *Alloprevotella* (−0.16), *Alistipes* (0.16), *Caldicoprobacter* (0.15) |
|  | C20:3N3 | *Pirellulaceae p-1088-a5 gut group* (0.29), *Anaeroplasma* (−0.22), *Alistipes* (0.21), *Coprococcus* (−0.21), *Romboutsia* (−0.19), *Terrisporobacter* (−0.18), *Alloprevotella* (−0.18), *Campylobacter* (−0.17), *Cerasicoccus* (0.17), *Prevotellaceae UCG-004* (0.17) |
|  | C20:3N6 | *Anaeroplasma* (0.37), *Succinivibrio* (0.33), *Alloprevotella* (0.24), unspecified Rikettsiales (0.21), *unspecified Ruminococcaceae* (−0.19), *Oscillospira* (−0.18), *Prevotella* (0.18), *Ruminococcus* (−0.15), *Oscillibacter* (−0.15), *Oscillospiraceae NK4A214 group* (−0.14) |
|  | C20:4 | *Bacteroidales p-2534-18B5 gut group* (−0.27), *Intestinibacter* (−0.22), *uncultured Paludibacteraceae* (−0.20), *Bradymonadales* (−0.19), *Anaerovorax* (0.19), *Anaeroplasma* (−0.19), *Phascolarctobacterium* (0.18), *Clostridium sensu stricto 1* (−0.18), *Erysipelatoclostridiaceae UCG-004* (−0.17), *undefined Peptostreptococcaceae* (−0.17) |
| *Longissimus dorsi* | C14:0 | *Anaerovibrio* (0.25), *Oribacterium* (0.21), *Clostridia vadin BB60 group* (−0.21), *unspecified Lachnospiraceae* (−0.20), *Bacteroidales F082* (−0.20), *Escherichia-Shigella* (−0.19), *Eubacterium nodatum group* (0.18), *Methanobrevibacter* (−0.17), *Ureibacillus* (0.17), *Oscillospiraceae NK4A214 group* (−0.16) |
|  | C15:0 | *Lachnospiraceae AC2044 group* (−0.27), *Coprococcus* (−0.24), *Campylobacter* (0.22), *Lachnospiraceae XPB1014 group* (−0.21), *undefined Lachnospiraceae* (−0.20), *Solobacterium* (0.19), *Bradymonadales* (0.19), *Anaerovibrio* (0.17), *Eubacterium coprostalinogenes group* (0.17), *Oligosphaeraceae horsej-a03* (−0.16) |
|  | C16:0 | *Clostridia UCG-014* (−0.26), *Rikenellaceae RC9 gut group* (0.25), *Oligosphaeraceae horsej-a03* (−0.21), *Colidextribacter* (0.21), *Eubacterium ruminantium group* (0.19), *Burkholderiales T34* (0.19), *Mucispirillium* (0.19), *Anaerorhabdus furcosa group* (0.19), *Monoglobus* (0.18), *Christensenellaceae R-7 group* (−0.18) |
|  | C17:0 | *Undefined Prevotellaceae* (0.29), *Prevotellaceae NK3B31 group* (0.26), *Pirellulaceae p-1088-a5 gut group* (0.24), *Erysipelatoclostridiaceae UCG-004* (−0.20), *Treponema* (−0.19), *Bacilli RF39* (−0.17), *Prevotella* (0.16), *unspecified Rikettsiales* (−0.16), *Lactobacillus* (0.16), *Colidextribacter* (−0.15) |
|  | C18:0 | *Unspecified Rikettsiales* (0.26), *Colidextribacter* (0.21), *Succinivibrio* (−0.21), *Agathobacter* (−0.20), *Prevotellaceae UCG-004* (0.20), *Rikenellaceae dgA-11 gut group* (0.19), *Escherichia-Shigella* (−0.17), *uncultured Prevotellaceae* (−0.17), *uncultured Ruminococcaceae* (0.17), *undefined Erysipelotrichales* (−0.17) |
|  | C20:0 | *Undefined Bacteroidales* (0.21), *Oscillospiraceae UCG-005* (0.21), *Eubacterium ruminantium group* (0.19), *Pirellulaceae p-1088-a5 gut group* (0.19), *Lachnospiraceae XPB1014 group* (0.18), *Eubacterium siraeum group* (−0.18), *Prevotellaceae UCG-003* (−0.17), *Terrisporobacter* (0.17), *Succinivibrio* (−0.16), *Bacteroidales dgA-11 gut group* (0.16) |
|  | C16:1 | *Prevotellaceae UCG-003* (0.26), *Anaerovibrio* (0.26), *Oscillibacter* (0.23), *undefined Bacteroidales* (−0.21), *uncultured Ruminococcaceae* (−0.21), *Sphaerochaeta* (0.17), *unspecified Lachnospiraceae* (−0.15), *unspecified Gastranaerophilales* (−0.15), *Treponema* (0.15), *Bacteroidales p-2534-18B5 gut group* (−0.15) |
|  | C17:1 | *Undefined Prevotellaceae* (0.24), *Prevotellaceae NK3B31 group* (0.24), *Bacilli RF39* (−0.20), *Erysipelatoclostridiaceae UCG-004* (−0.18), *Lachnospiraceae UCG-007* (−0.18), *Treponema* (−0.18), *Mucispirillum* (0.17), *Bacteroidales p-2534-18B5 gut group* (−0.17), *Mycoplasma* (0.16) |
|  | C18:1 | *Rikenellaceae RC9 gut group* (−0.28), *unspecified Rikettsiales* (−0.22), *Colidextribacter* (−0.21) *Clostridia UCG-014* (0.20), *Oligosphaeraceae horsej-a03* (0.19), *Succinivibrio* (0.19), *Eubacterium ruminantium group* (−0.18), *Monoglobus* (−0.18), *Escherichia-Shigella* (0.17), *Alistipes* (0.17) |
|  | C18:2 | *Oscillibacter* (−0.24), *Bacteroidales F082* (0.23), *Helicobacter* (−0.22), *Ureibacillus* (−0.22), *Intestinibacter* (−0.19), *Clostridia UCG-014* (0.18), *Terrisporobacter* (−0.18), *Clostridium sensu stricto 1* (−0.18), *Blautia* (−0.17), *Hydrogenophilus* (−0.17) |
|  | C18:3 | *Intestinibacter* (−0.25), *Helicobacter* (−0.24), *undefined Erysipelotrichales* (−0.23), *Oscillibacter* (−0.22), *Bacilli RF39* (0.21), *Ureibacillus* (−0.18), *Anaerovoracaceae Family XIII AD3011 group* (0.18), *Bacteroidales F082* (0.18), undefined Alphaproteobacteria (0.17), *Mycoplasma* (0.16) |
|  | C20:1 | *Lachnospiraceae AC2044 group* (0.22), *Anaerovibrio* (−0.20), *Eubacterium halii group* (0.19), *Succinivibrio* (−0.19), *Bradymonadales* (−0.19), *Cerasicoccus* (0.17), *Alloprevotella* (−0.17), *Lachnospiraceae XPB1014 group* (0.17), *Colidextribacter* (0.16), *Eubacterium nodatum group* (0.16) |
|  | C20:2 | *Uncultured Erysipelatoclostridiaceae* (0.32), *uncultured Ruminococcaceae* (−0.19), *Anaerovibrio* (0.18), *Oligosphaeraceae horsej-a03* (−0.16), *Ruminococcus* (−0.15), *Mucispirillum* (0.15), *unspecified Ruminococcaceae* (−0.15), *Phascolarctobacterium* (0.15), *Prevotellaceae UCG-004* (0.14), *Methanobrevibacter* (0.14) |
|  | C20:3N3 | *Alistipes* (−0.23), *Rikenellaceae dgA-11 gut group* (0.21), *Lachnospiraceae UCG-007* (−0.19), *Lachnospiraceae XPB1014 group* (0.19), *Akkermansia* (0.18), *Eubacterium siraeum group* (−0.18), *Anaerovibrio* (−0.18), *Roseburia* (−0.17), *Oligosphaeraceae horsej-a03* (0.17), *uncultured Coriobacteriales* (−0.17) |
|  | C20:3N6 | *Campylobacter* (0.28), *Bacteroides* (0.26), *Solobacterium* (−0.25), *Desulfovibrio* (0.22), *Prevotellaceae UCG-001* (0.20), *Prevotella* (−0.19), *unspecified Lachnospiraceae* (0.18), *unspecified Muribaculaceae* (−0.18), *Rikenellaceae dgA-11 gut group* (0.18), *undefined Clostridia* (0.16) |
|  | C20:4 | *Oscillibacter* (−0.26), *Bacteroidales F082* (0.21), *uncultured Paludibacteraceae* (−0.21), *Oribacterium* (−0.19), *Prevotellaceae UCG-003* (0.18), *Pirellulaceae p-1088-a5 gut group* (−0.18), *Lachnospiracecae XPB1014 group* (0.17), *unspecified Bradymonadales* (−0.17), *Lachnospiraceae NK4A136 group* (0.16), *undefined Peptostreptococcaceae* (−0.16) |
| Rectal content | Acetic | *Akkermansia* (0.20), *Prevotellaceae NK3B31 group* (−0.20), *Prevotella* (−0.20), *Escherichia-Shigella* (0.19), *Christensenellaceae R-7 group* (0.19), *undefined Spirochaetaceae* (0.19), *Oscillospiraceae NK4A214 group* (0.19), *Oscillospirales UCG-010* (0.18), *Desulfovibrio* (0.18), *Mycoplasma* (−0.18) |
|  | Propionic | *Chrstensenellaceae R-7 group* (−0.26), *Prevotellaceae UCG-001* (0.24), *Oscillospirales UCG-010* (−0.23), *Clostridium sensu stricto 1* (−0.22), *Terrisporobacter* (−0.21), *Alloprevotella* (0.21), *Oscillospiraceae NK4A214 group* (−0.18), *Mycoplasma* (0.18), *Sphaerochaeta* (0.17) |
|  | n-Butyric | *Prevotella* (0.26), *Clostridia vadin BB60 group* (−0.20), *Akkermansia* (−0.20), *Oscillospiraceae NK4A214 group* (−0.19), *unspecified Muribaculaceae* (0.18), *Prevotellaceae NK3B31 group* (0.18), *Cerasicoccus* (−0.18), *Solobacterium* (0.17), *Methanobrevibacter* (−0.16), *Oscillospirales UCG-010* (−0.16) |
|  | iso-Butyric | *Oscillospiraceae UCG-002* (0.28), *Bacteroidales F082* (0.21), *Rikenellaceae dgA-11 gut group* (0.19), *Oscillospiraceae UCG-005* (−0.18), *Oscillospirales UCG-010* (0.17), *Streptococcus* (0.17), *Ureibacillus* (−0.16), *Clostridia vadin BB60 group* (0.16), *Kiritimatiellae WCHB1-41* (0.16), *Frisingicoccus* (0.15) |
|  | n-Valeric | *Methanobrevibacter* (0.21), *Oscillospiraceae NK4A214 group* (0.20), *Terrisporobacter* (−0.19), *Bacteroidales F082* (0.18), *Oscillibacter* (−0.18), *Lachnoclostridium* (−0.18), *Roseburia* (−0.18), *Christensenellacecae R-7 group* (0.17), *Oscillospiraceae UCG-002* (0.17), *Prevotella* (−0.17) |
|  | iso-Valeric | *Oscillospiraceae UCG-002* (0.28), *Bacteroidales F082* (0.21), *Subdoligranulum* (−0.19), *Christensenellaceae R-7 group* (0.19), *Oscillospirales UCG-010* (0.18), *Burkholderiales T34* (−0.18), *Methanobrevibacter* (0.18), *Clostridia vadin BB60 group* (0.17), *Roseburia* (−0.17), *Rikenellaceae dgA-11 gut group* (0.16) |
